# The impact of nursing staff education on diabetes inpatient glucose management: a pilot cluster randomised controlled trial

**DOI:** 10.1186/s12902-022-00975-y

**Published:** 2022-03-10

**Authors:** Milan K. Piya, Therese Fletcher, Kyaw P. Myint, Reetu Zarora, Dahai Yu, David Simmons

**Affiliations:** 1 School of Medicine, Western Sydney University, Campbelltown, NSW Australia; 2 Macarthur Diabetes Endocrinology and Metabolism Service, Camden and Campbelltown Hospitals, Campbelltown, NSW Australia; 3 Primary Care Centre Versus Arthritis, School of Medicine, Keele University, Staffordshire, UK

**Keywords:** Diabetes, HCP education, Online learning, Inpatient diabetes, Healthcare delivery, Hypoglycaemia

## Abstract

**Background:**

An increasing number of patients in hospital have diabetes, with most of them cared for by non-specialist staff. The effect of diabetes education for staff on patient outcomes, as well as the most effective method of staff education is unclear. Therefore, the aim of this study was to compare diabetes outcomes in medical wards where nursing staff were offered one face-to-face (F2F) session followed by access to online education (online), F2F education only, or standard care (control).

**Methods:**

We conducted a pilot cluster randomised controlled trial involving 16-weeks baseline/rollout followed by a 28-week post-intervention period across three medical wards (clusters) in a Sydney Teaching Hospital. The online ward provided an online competency-based diabetes education program and 1-h F2F teaching from a diabetes nurse educator (DNE), the F2F ward provided four separate 1-h teaching sessions by a DNE, with no additional sessions in the control ward. The primary outcome was length of stay (LOS); secondary outcomes included good diabetes days (GDD), hypoglycaemia and medication errors. Poisson and binary logistic regression were used to compare clusters.

**Results:**

Staff attendance/completion of ≥ 2 topics was greater with online than F2F education [39/48 (81%) vs 10/33 (30%); *p* < 0.001]. Among the 827/881 patients, there was no difference in LOS change between online [Median(IQR) 5(2–8) to 4(2–7) days], F2F [7(4–14) to 5(3–13) days] or control wards [5(3–9) to 5(3–7) days]. GDD improved only in the online ward 4.7(2.7–7.0) to 6.0(2.3–7.0) days; *p* = 0.038. Total patients with hypoglycaemia and appropriately treated hypoglycaemia increased in the online ward.

**Conclusions:**

The inclusion of online education increased diabetes training uptake among nursing staff. GDD and appropriate hypoglycaemia management increased in the online education wards.

**Trial registration:**

Prospectively registered on the Australia New Zealand Clinical Trials Registry (ANZCTR) on 24/05/2017: ACTRN12617000762358.

**Supplementary Information:**

The online version contains supplementary material available at 10.1186/s12902-022-00975-y.

## Introduction

The increasing prevalence of diabetes places a huge burden on health systems, with one in every 4 or 5 patients in hospital having diabetes [1–3]. People with diabetes have higher mortality, hospital readmission as well as complications including hypoglycaemia and infections [3–6]. The resulting increased length of hospital stay has both financial as well as hospital capacity implications [6, 7]. Although studies have shown a reduced hospital readmission rate and hospitalisation cost when patients are looked after by a specialist inpatient diabetes team rather than the primary service team that includes ward-based nurses [8], the increase in number of inpatients with diabetes means that the majority of care provided to people with diabetes in hospital is not provided by diabetes specialist staff, making diabetes education for non-specialist nursing staff a priority [9]. Errors in medication, in particular insulin, can result in poor diabetes control in hospital including hypoglycaemia [1, 3, 10]. One previous way of measuring diabetes management in hospital has been with the use of good diabetes days (GDD) based on the (NaDIA, UK) definition [1], where a GDD was a day where there were no hypos and one or less episodes of hyperglycaemia.

However, healthcare practitioners face difficulty in accessing face to face education due to understaffing, workplace demands, personal commitments, expense and variation in individual learning needs [11]. Previous studies have shown a low confidence in diabetes management among healthcare professionals in hospital including junior doctors, nursing staff and pharmacists [12]. Attendance at traditional face to face continuing professional development (CPD) programs does have advantages but usually involve days away from clinical duties, and often involve travel and additional cost [13]. For time poor busy health professionals, particularly ward nursing staff, online programs are appealing and offer similar positive effect on learning compared to traditional face to face learning programs [14]. A range of hospital ward staff education approaches are available both face to face and online [15], but none have been rigorously evaluated in randomised controlled trials.

The purpose of this pilot study was to evaluate uptake of an online competency-based program among ward-based nursing staff, followed by a comparison of reach and clinical outcomes in a pilot cluster randomised controlled trial.

## Subjects, methods and materials

### Formative evaluation

The online competency based program used was an Australian [16] adaptation of the Cambridge Diabetes Education Program (CDEP), itself underpinned by the UK national diabetes competencies frameworks [9]. The program contains multiple choice questions, linkages to national and international learning resources, and level progression through 100% mastery. The program was made available to all staff on a single medical ward in a 306-bed tertiary hospital in Sydney, Australia in 2016, which was a different ward to the three wards in the cluster randomised trial, but was similar in terms of nursing staff numbers and nurse to patient ratios on the ward. Staff were invited by email and information sessions to voluntarily undertake two online modules (Suppl table 1): “*What is Diabetes”* and “*Managing Diabetes in Hospital”* over a 6-week period. Following each module, an embedded evaluation score graded 1–5 (1 = much worse, 2 = worse, 3 = same, 4 = better, 5 = much better) was completed regarding increase in overall competency in diabetes patient care, increase in overall confidence with managing patients with diabetes, and an increase in familiarity with diabetes guidelines in relation to managing patients with diabetes. Formative evaluation included three focus groups among participants undertaking the online modules which were recorded, transcribed verbatim and de-identified. Thematic analysis was used to identify and group themes [17].

While no formative evaluation was conducted for the F2F teaching ward, feedback was taken into account from previous F2F teaching sessions on the wards and discussions with the nurse unit manager. Based on this feedback, the F2F teaching was arranged to be conducted on the ward itself rather than a lecture theatre, and the time of the session was chosen by the nurse unit manager to allow for the maximum number of nursing staff to be available to attend the session.

### Pilot cluster randomised controlled trial (CRCT)—study design and intervention

**Fig. 1:**
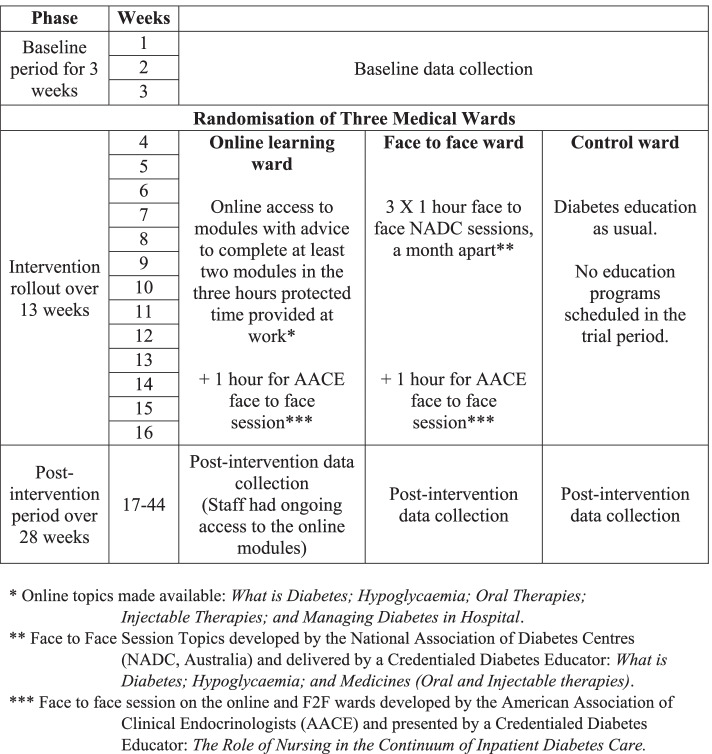
Study timeline and interventions on the three randomised wards

This was a pilot cluster randomised controlled trial (CRCT) (ACTRN12617000762358) on three medical wards in the same hospital in Sydney, Australia. Each medical ward was a cluster, and randomised to receive either (1) one hour of protected time on three occasions with access to complete online education modules followed up by 1-h of face to face education for a total of four hours (online), (2) face-to-face (F2F) education for four 1-h sessions with protected time for a total of four hours, or (3) staff diabetes education as usual (control) (Fig. 1). Randomisation was undertaken through a sealed envelope delivered through an independent person. All investigators except TF were blinded to the allocation until after analyses were completed. Each ward included 26–30 beds, with 33–48 nurses. From June 2017, as shown in Fig. 1, baseline data was collected over three weeks and the three-month roll out period when education was delivered/made available. Post-intervention data were collected for the subsequent six months. Ethics approval was granted by the South Western Sydney Local Health District HREC (formative evaluation: LNR/16/LPOOL/503; CRCT: HE17/047).

### Participants and data collection

A daily census was conducted of all inpatients with pre-existing diabetes on the three wards. Length of stay was calculated post-discharge, and electronic medical records were reviewed by a blinded researcher using a pre-specified audit form, based on the National Diabetes Inpatient Audit (NaDIA, UK) [1]. Glucose data, hypo and hyperglycaemia episodes and medication errors were extracted from paper charts, with confirmation from patient notes. Diabetes medication errors included wrong medication charted or given; prescription not signed for, wrong dose charted or given, correct medication or dose not charted, correct medication charted but at the wrong time, as well as charted medication not give at the appropriate time. If patients moved between wards, patient data were analysed by the ward where they were first identified by the daily census.

### Intervention

Nursing staff on the online ward were given access to the online modules with advice to complete at least two modules in three hours of protected time in an office with a computer away from the clinical environment, during work hours, as shown in Fig. 1. This was followed by one face to face session on *The Role of Nursing in the Continuum of Inpatient Diabetes Care* developed by the American Association of Clinical Endocrinologists (AACE), at the end of the rollout phase. Staff had ongoing access to the online modules. On the F2F ward, nursing staff were provided with three separate one-hour face to face sessions over three months, developed by the National Association of Diabetes Centres (NADC, Australia), plus the hour long AACE session identical to the online ward. The control ward had no scheduled diabetes education sessions in the intervention period.

### Outcomes

The primary outcome measure was the difference between wards in the change in length of stay. Secondary outcomes are shown in Fig. 1 including good diabetes days (GDD) based on the (NaDIA, UK) definition [1], where a GDD was a day where there were no hypos and one or less episodes of hyperglycaemia. Hypos were defined as documented capillary glucose ≤ 4.0 mmol/L, severe hypos as documented capillary glucose ≤ 3.0 mmol/L and hyperglycaemia as ≥ 11.0 mmol/L. GDD was adjusted to a standardised 7-day admission, so that the outcome was measured as GDD per week. For admissions less than 7 days, GDD = (number of good diabetes days/LOS) *7.

#### Statistical analysis

As a pilot study, no power calculations were undertaken and the duration of the study (and ergo the sample size of 400) was defined pragmatically. We predicted that it would take three months to undertake the study but roll out was delayed for administrative reasons, so this actually took 6 months. An intention to treat (ITT) analysis of the inpatient data was undertaken by a blinded statistician in the UK (DY). Analysis of variance (ANOVA) was used to detect differences in baseline characteristics between clusters (wards) and between the baseline/rollout and intervention period. Poisson regression and binary logistic regression analysis were used to analyse the change in primary and secondary outcomes including of lengths of stay (LOS) and good diabetes days, adjusting for clustering and baseline differences. STATA MP15.1 (Stata Corporation, College Station, TX, USA) was used for all analyses. Tests are 2-tailed and *P* < 0.05 was considered statistically significant.

## Results

### Formative evaluation

Before the CRCT, formative evaluation was undertaken on a separate ward that was similar in nursing staff numbers and patient bed numbers, to ensure acceptability of the online program. Overall, 18/33 ward nursing staff registered for the online program, of whom nine completed two topics, one completed one topic and eight nurses started but did not complete any topics. Thirteen ward staff members voluntarily participated in three focus groups convened between 13 December 2016 and 2 February 2017, lasting between 27–32 min. Four themes emerged in the formative evaluation as shown in Supplementary Table 1 (see Supplementary Table 2 for quotes):

- Perceptions and Experience
- Functionality of program/webpage
- Contextual and Clinical Relevance
- Perceived Access: Barriers and Facilitators

Staff self-reported an increase in overall competency in diabetes patient care mean score of 4.4/5, an increase in overall confidence with managing patients with diabetes mean score of 4.3/5, and an increase in familiarity with diabetes guidelines in relation to managing patients with diabetes mean score of 4.3/5. Adjustments to the program were made following the formative evaluation (Suppl table 1).

### Pilot cluster randomised controlled trial

#### Reach

Uptake of diabetes education was greater on the online ward compared to the F2F ward; 90% (*n* = 43/48) nurses completed one diabetes topic compared to 45% (*n* = 15/33) on the F2F ward (*p* < 0.01). Two diabetes topics were completed by 81% (*n* = 39/48) on the online ward compared to 30% (*n* = 10/33) on the F2F ward (*p* < 0.001). Of the 43 nurses completing at least one topic in the online ward, only nine (19%) attended the face to face teaching session on that ward.

##### Embedded online evaluation survey—CRCT participants

Staff from the online ward self-reported an increase in overall competency in diabetes patient care- mean score of 4.1/5, an increase in overall confidence with managing patients with diabetes- mean score of 4.0/5, and an increase in familiarity with diabetes guidelines in relation to managing patients with diabetes- mean score of 4.0/5.

##### Baseline patient data

**Fig. 2:**
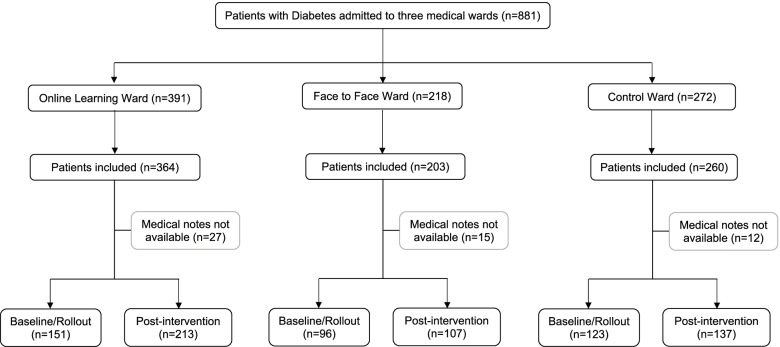
Consort Diagram

Of the 881 eligible patients, 827 were included in the trial as shown in the Consort diagram (Fig. 2). The overall mean age of patients in the CRCT wards was 69 ± 12.4 years, with 46.1% female, and 73.3% of European descent. (Other population groups included 3% Aboriginal, 8.2% Pacific Islanders, 6.2% Asians, 9.3% others). Over 85% of the patients had T2DM, most were emergency admissions, and a quarter of patients were using insulin on admission. Table 1 shows no significant difference in characteristics between wards at baseline, or between baseline/rollout vs post-intervention period apart from during the post-intervention phase on the online ward where there was a change in mix of elective admissions and transfers (*p* = 0.01), less diet/more medication treated patients (*p* = 0.012), more hyperglycaemia (*p* < 0.001) and more patients with macrovascular disease (*p* < 0.001). The F2F ward had less patients admitted for a diabetes related reason in the post-intervention period (*p* = 0.044).

**Table 1:** Pilot CRCT Demographic data

**Medical Wards** **Phase**	**Online Learning Ward**		**P**_**B**_	**Face to Face (F2F) Ward**		**P**_**F**_	**Control Ward**		**P**_**C**_	**P**_**baseline**_
	**Baseline + Rollout**	**Post intervention**		**Baseline + Rollout**	**Post intervention**		**Baseline + Rollout**	**Post intervention**		
Number of patients (n)	151	213		96	107		123	137		
Age in years	69.8 (± 12.7)	68.4 (± 11.2)	0.446	68.2 (± 12.0)	67.1 (± 11.2)	0.773	70.9 (± 13.2)	69.9 (± 13.7)	0.581	0.098
Gender (Female)	56(37%)	88(41%)	0.330	54(56%)	49(46%)	0.078	69(56%)	65(47%)	0.325	0.968
European Descent	115(76%)	143(67%)	0.062	75(78%)	81(76%)	0.818	92(75%)	100(73%)	0.893	0.471
**Diabetes Type**										
Type 1 (%)	4(3%)	11(5%)	0.182	3(3%)	7(7%)	0.295	8(7%)	4(3%)	0.259	0.232
Type 2 (%)	147(97%)	202(95%)		93(97%)	99(93%)		115(93%)	133(97%)		
Glucose on admission (mmol/L)	12.5(± 5.3)	13.0(± 5.1)	0.494	13.9(± 7.0)	12.6(± 5.1)	0.200	13.4(± 6.1)	13.3(± 5.3)	0.900	0.734
**Type of admission**										
Emergency	141(93%)	196(92%)	0.011	90(94%)	95(89%)	0.414	121(98%)	132(96%)	0.071	0.488
Elective	1(1%)	12(6%)		1(1%)	2(2%)		0(0%)	3(2%)		
Transfer	9(6%)	5(2%)		5(5%)	10(9%)		2(2%)	2(1%)		
**Treatment on Admission**										
Lifestyle	18(12%)	9(4%)	0.012	11(11%)	9(8%)	0.244	13(11%)	18(13%)	0.189	0.690
Tablet	86(57%)	128(60%)		45(47%)	66(62%)		58(47%)	75(55%)		
Insulin	39(26%)	66(31%)		30(31%)	25(23%)		32(26%)	37(27%)		
GLP 1	0(0%)	4(2%)		0(0%)	3(3%)		0(0%)	3(2%)		
Insulin treated T2DM (%)	35(23%)	55(26%)	0.319	27(28%)	18(17%)	0.074	24(20%)	33(24%)	0.561	0.428
Total patients with BGL > 11 mmol/L	70(46%)	124(58%)	< 0.001	56(58%)	60(56%)	0.792	69(56%)	82(60%)	0.703	0.814
**Admission reason**										
Diabetes	3(2%)	3(1%)	0.976	6(6%)	1(1%)	0.044	12(10%)	6(4%)	0.110	0.014
Other	147(97%)	210(99%)		90(94%)	106(99%)		111(90%)	131(96%)		
**Documented Complications present**										
Chronic kidney disease	9(6%)	18(8%)	0.382	2(2%)	2(2%)	0.944	9(7%)	13(9%)	0.334	0.228
Foot disease	5(3%)	8(4%)	0.830	2(2%)	4(4%)	0.457	7(6%)	7(5%)	0.624	0.308
Macrovascular	22(15%)	64(30%)	< 0.0001	13(14%)	20(19%)	0.779	15(12%)	18(13%)	0.349	0.641
Autonomic	3(2%)	13(6%)	0.132	3(3%)	4(4%)	0.770	3(2%)	7(5%)	0.960	0.832
Eye disease	3(2%)	9(4%)	0.481	1(1%)	2(2%)	0.602	2(2%)	6(4%)	0.139	0.641

P_baseline_ is the *p* value for the difference between baseline values for the three wards (clusters). P_B_ is *p* value for the difference between baseline/rollout and post-intervention in online learning ward, and similarly P_F_ and P_C_ are the *p* values for F2F and control wards

##### Outcomes for the CRCT

Table 2 shows no differences in hospital length of stay. The online ward, but not the F2F or control wards showed increased number of good diabetes days (GDD) from median (IQR) 4.7 (2.7–7.0) at baseline/rollout to 6.0 (2.3–7.0) in the post-intervention phase (*p* = 0.04), along with increased hypos treated appropriately by ward staff (80% vs 85%, *p* = 0.026) and total hypos (10% vs 16%, *p* = 0.043), while severe hypos was borderline significant (3% vs 6%, *p* = 0.05). In the control ward alone, there was a reduction of the proportion of hypos being appropriately treated by ward staff from 88 to 68% in the post-intervention phase (*p* = 0.028), and an increase in the number of total medication errors in the control ward from 9 to 20% (*p* = 0.0028).

**Table 2:** Effect of Intervention on Length of Stay, Glycaemic Control and Quality of Care

**Medical Wards**	**Online Learning Ward**		**P**_**B**_	**Face to Face (F2F) Ward**		**P**_**F**_	**Control Ward**		**P**_**C**_	**P**_**baseline**_
**Phase**	**Baseline + Rollout**	**Post inter-vention**		**Baseline + Rollout**	**Post inter-vention**		**Baseline + Rollout**	**Post inter-vention**		
Number of patients (n)	151	213		96	107		123	137		
Length of stay – median (IQR)	5.00 (2.0–8.0)	4.00 (2.0–7.0)	0.4084	7.00 (4.0–14.0)	5.00 (3.0–13.0)	0.0964	5.00 (3.0–9.0)	5.00 (3.0–7.0)	0.0894	0.0007
Good diabetes days (GDD)/week – median (IQR)	4.66 (2.7–7.0)	6.00 (2.3–7.0)	0.0375	5.25 (2.8–7.0)	5.72 (3.5–7.0)	0.5262	4.2 (2.3–6.0)	5.25 (2.3–7.0)	0.0780	0.1503
Total patients with hypos	15(10%)	34(16%)	0.0426	16(17%)	17(16%)	0.9311	16(13%)	19(14%)	0.8272	0.1794
Total severe hypos (< 3.0 mmol/L)	5(3%)	13(6%)	0.050	5(5%)	5(5%)	0.854	3(2%)	9(7%)	0.144	0.644
Hypos treated appropriately (patient %)	12(80%)	29(85%)	0.026	15(94%)	16(94%)	0.319	14(88%)	13(68%)	0.028	< 0.0001
Total medication errors	23(15%)	35(16%)	0.938	14(15%)	12(11%)	0.938	11(9%)	27(20%)	0.0028	0.058

P_baseline_ is the *p* value for the difference between baseline values for the three wards (clusters). P_B_ is *p* value for the difference between baseline/rollout and post-intervention in the online learning ward, and similarly P_F_ and P_C_ are the *p* values for F2F and control ward

## Discussion

This pilot study is the first randomised trial to evaluate the impact of non-specialist nursing staff education on patient outcomes, and directly compare uptake of online learning with traditional F2F education. The study demonstrated a significantly greater uptake of online learning compared to F2F learning, accompanied by a self-reported improvement in confidence and competency in diabetes patient care as well as an increased familiarity with diabetes guidelines. The primary outcome of the pilot CRCT of length of stay did not differ between the wards. However, there was an increase in the number of GDD/week in the online ward only.

Computer based and online learning tools for staff education have been in place for a number of years, with increasing accessibility with smart phones and handheld devices [13]. Studies have shown an improvement in healthcare professional confidence in managing diabetes with face to face education in hospital [12, 18, 19]. While online learning complemented by face to face education (blended online learning) has been shown in a recent meta-analysis to demonstrate consistently better effects on knowledge outcomes compared to traditional learning in health education [20], this has not been shown in studies in diabetes. However, the advantage of being able to complete online CPD modules at a time and place convenient to you does have advantages, but also means that staff need the motivation and time to complete these modules.

In this study, the face to face sessions were conducted on the study ward at a time agreed with the ward nurse unit manager to make it easily accessible for staff to attend. In spite of this ease of access, staff not being on shift on the specified days and some staff not being able to attend the sessions due to clinical needs on the wards meant the attendance rate for the four education sessions was < 50%. This is in contrast to the 90% completion of one module and 81% completion of two modules in the online ward. Even in the online ward, < 20% attended the single face to face teaching session provided. Formative thematic analysis showed that main themes as seen in Suppl Table 1 and 2 include perceptions and experience, functionality of program, contextual and clinical relevance and perceived access barriers and facilitators. This was used to help modify the online delivery of the modules, and also the addition of a face to face component to make it blended online learning, as well as agreement to allow protected time at work. These themes on barriers are similar to those in a recent qualitative evaluation of nursing teams on using iPad delivered diabetes education, where the four themes that emerged were educational program and content, platform usability, tablet feasibility and workflow considerations [21]. Addressing these themes in the pilot phase formative evaluation before rollout of the Pilot CRCT may have helped completion rates and uptake. Also, with all the staff on the ward asked to complete the modules, there was opportunity for peer learning and support to encourage each other to complete these modules and improve the care of patients with diabetes.

Length of stay (LOS) was the primary outcome of the study but this was not affected by staff education in this study. This may not have been too surprising as LOS is affected by a multitude of factors beyond diabetes such as acuity of the co-morbidity, social circumstances, availability of the destination and overall efficiency of the inpatient care [22]. Hence, while a whole systems approach to managing inpatient diabetes, the Diabetes Inpatient Care and Education (DICE) initiative did reduce length of stay in people with diabetes [23], a randomised study in Australia with an early diabetes team intervention did not show changes in length of stay [4]. Interestingly, that study also showed a reduction in adverse glycaemic days (capillary glucose ≤ 4.0 mmol/L or ≥ 15.0 mmol/L) in the post-intervention phase.

On the other hand, good diabetes days/week (GDD/week) is a direct measure of diabetes control in hospital and has been used to describe glycaemic control in hospital in the UK National Diabetes Inpatient Audit [1]. With over 800 patients in this CRCT, the improvement in GDD/week in the online ward, but not the F2F ward or control ward suggests that an increased uptake and completion rate of competency-based diabetes education by nursing staff can improve glycaemic control in hospital. Previous studies have suggested an increased risk of infections in people with diabetes admitted to hospital [4], and improved glycaemic control in hospital has the potential to reduce this, although this was not measured in this study. It was interesting to find that in spite of an increase in the number of GDD/week, there was a statistically significant increase in hypos and severe hypos in the online ward between the baseline/rollout phase and post-intervention phase. These actual numbers were relatively small and the post-intervention percentages seemed to be closer to the values in the other wards for both durations and comparable to the UK National Diabetes Inpatient Audit data that showed 18.4% of patients had an episode of hypo in the previous 7 days [1]. It is known that hypoglycaemia in hospital can be asymptomatic and often goes undetected [24, 25]. One potential explanation for the increase in hypos detected in this study could be that the nursing staff were involved in increased vigilance, testing and documentation in the online ward that led to increased testing and detection of hypos, severe hypos and hyperglycaemia, whereas some of the patients developing hypo symptoms were given food or hypo corrections without testing or documenting the capillary blood glucose level.

Hypos are a major problem in people with diabetes in hospital and have been linked to greater length of stay in hospital as well as in-hospital mortality [5, 26]. However, in this study, length of stay did not seem to be affected by the increase in hypos on the online ward. However, of the hypos treated, there was an increase in the number of hypos treated appropriately in the online ward, which was encouraging and this is probably a direct effect of the online learning of nursing staff who are critical in the recognition, documentation and management of hypoglycaemia on the ward [27]. Worryingly, there was a reduction in the number of hypos treated appropriately in the control ward. This was also accompanied by an increase in medication errors in the control ward only, which in addition to errors in prescription included inappropriate omissions or delays in administering medications including insulin. While these results are surprising and unexpected, this may be a result of nursing staff being less engaged in diabetes care compared to the other wards due to not being offered any diabetes education. Many hospitals have come up with functionalities on both electronic and paper-based insulin prescription systems to reduce errors, but approaches, terminology and outcome measures have not been standardised as shown in a recent systematic review, which makes it difficult to compare the various interventions [28].

One of the ways to improve diabetes care in hospital has been use of technology including networked blood glucose meters with a dashboard or the use of Flash Glucose continuous glucose monitoring systems [29]. The use of Flash Glucose found a greater use during the Covid-19 pandemic where staff did not have to perform capillary blood glucose readings [30]. There is also potential for closed loop insulin delivery systems being used in hospital in the near future, which reduces the potential for human error [31]. However, this would still need ward staff to support patients and help them. If anything, this technology could identify which wards or staff needed education. The extent of the increase in uptake in staff education in this study through the additional access to online learning, and the associated improved GDD and hypo management, suggests the importance of such educational programs when social distancing limits the capacity to provide or attend face to face education sessions.

The lack of difference in medication errors in the online or F2F wards may be linked to the fact that the education was limited to nursing staff and not medical staff, who are the ones prescribing the medications including insulin. The fact that medical doctors and teams cover more than one ward, especially when providing out of hours cover, made it too difficult to randomise them into groups in this study. The lack of difference in LOS in the same way, may be because often the decision to discharge is led by the medical team.

The strengths of this study are that this is the first RCT assessing the effect of nursing staff education on patient outcomes with such a large number of patients over 6 months. The RCT was implemented robustly, with unbiased randomisation, blinding of the investigators and of the statistician. Also, the online learning modules were modified following formative evaluation from staff, before the CRCT. The main limitations of this study are that the baseline planned phase of 2 weeks had to be extended to 3 weeks’ baseline and 13 weeks’ rollout phase to allow more time for both the face to face education sessions to be organized for the ward nursing staff, as well as more protected time to be allowed for the online ward-based nursing staff. This delay in implementation is perhaps a sign that nursing education, whether it be online or face to face, was a low priority even in this CRCT setting. Another limitation is that we did not have the demographic data for the nursing staff involved in the study. However, the wards were chosen for the study as they were all medical wards of a similar size with a similar number of nursing staff and patient beds, and they were randomised after the start of the trial. We were also unable to include patient reported outcomes or patient satisfaction data from these wards.

In conclusion, the addition of access to online learning as well as face to face to education, significantly increased uptake of diabetes education among hospital non-specialist nursing staff. Although length of stay was not reduced, glycaemic control and hypoglycaemia treatment were improved. The findings of this pilot study suggest that there would likely be further benefits if online learning was rolled out to include medical staff, which, with social distancing and meeting restrictions amidst the Covid-19 pandemic, may offer a better alternative to traditional methods of diabetes education. Further randomised clinical trials to confirm these findings are required.

## Supplementary Information

**Additional file 1.** 

## Data Availability

The datasets used and/or analysed during the current study are not publicly available as they include patient clinical data in the hospital, but some of the data are available from the corresponding author on reasonable request.
