# Supplementary material for: The impact of nursing staff education on diabetes inpatient glucose management: a pilot cluster randomised controlled trial
Source: BMC Endocr Disord. 2022 Mar 10;22:61. doi: 10.1186/s12902-022-00975-y (PMC8911103; doi:10.1186/s12902-022-00975-y)
Supplement: Supplementary file 1 — Additional file 1. [file 12902_2022_975_MOESM1_ESM.docx]

**The Impact of Nursing Staff Education on Diabetes Inpatient Glucose Management: A Pilot Cluster Randomised Controlled Trial**

Supplementary Table 1:

Topics included in the Australian adaptation of the Cambridge Diabetes Education Program

| Topics Made Available:  Module 1: What is Diabetes  Module 2: Hypoglycemia  Module 3: Oral Therapies  Module 4: Injectable Therapies  Module 5: Managing Adults with Diabetes in Hospital |
| --- |
| Adjustments to online program following the formative evaluation:  Increased clinical accuracy  Increased Australian clinical relevance and terminology  Reduction in quiz series length and answer options  Increased certification of continuing professional development (CPD) time awarded  Nursing staff were also provided with one hour face to face session to make it blended online learning, and provided protected time at work to complete these modules. |
| Thematic analysis results in the formative evaluation of the online program  (see supplemental table 2 for quotes).  1. Perceptions and Experience   - Beneficial in terms of knowledge and impact on diabetes clinical practice - Thought to be ‘very well done’ and ‘brilliant’ and thought ‘it should be made compulsory’ as it ‘filled a gap in education.’ - ‘Difficult’, ‘too hard’ and ‘dense’ for ward staff, and thought the level more suitable for diabetes specialist nurses or for staff working on an ‘endocrine ward’. - Required a lot of reading. - The learning experience could have been enhanced by offering diabetes information and resources prior to undertaking the quiz. - Met the same competence levels relevant to their work.   2. Functionality of program/webpage   - ‘Frustrating’ in relation to getting one answer incorrect in quizzes that offered numerous answer options. - Often resulted in repeatedly redoing the quiz in order to achieve 100% mastery. - Was ‘time consuming’ and made staff feel ‘disappointed in getting things wrong’. - The webpage was easy to navigate. - Occasionally correct answers were thought marked as incorrect - Occasionally resources not opening, especially when they undertook the program whilst in hospital (where a firewall could block web-based resources opening).   Participants suggested   - Offering less options per question, so as not to repeatedly redo the questions and make it harder to get wrong. - Needed more acknowledgement for the time spent doing the topics as it took longer than expected.   3. Contextual and Clinical Relevance   - Despite the pharmacology being hard, the medication sections were ‘helpful’. - The program’s resources to be ‘great’ and liked the way the ‘resources were graded’. - The terminology needed to be more localized noticing a ‘British thread’ throughout. - Hypoglycemia treatment and some insulins included were different to those used locally and needed to be amended.   4. Perceived Access Barriers and Facilitators   - Finding time within work and out of work was difficult because of competing and unpredictable clinical workload and family /home commitments. - computers were limited on the ward and clinical purposes would always override computer use for learning. Consequently, fully engaging and learning when doing the program proved difficult because of interruptions, time of day (evening or night) and being too tired. - Staff believed the content to be important, but time needed to be provided to staff to undertake and focus on the program during work. |

Supplementary Table 2: Participant responses relating to individual themes in the formative evaluation over three focus groups (FG) with nursing staff

| 1. ***Perceptions and Experience***   *Overall I felt the course was brilliant. Participant Focus Group 1 (FG1)*  *It’s a really good program. Participant Focus Group 3 (FG3)*  *I really enjoyed doing it. (FG3)*  *I think it’s done very well. (FG1)*  *I said it was too much, but as I continued doing it, I really got interested. (FG3)*  *The other thing I found though with this kind of stuff was that the gaps in education, like for me personally. (FG1)*  *Can we make it as compulsory? We need to do that. (FG3)*  *So someone is having a hypo in the mornings when we check it, the night-time …. needs to be looked at. (FG3)*  *I can make a judgement now when to call the RMO ....and feel comfortable about giving insulin’ (FG3)*  *Someone was being discharged and it was after hours, that patient was going home with insulin and not taught how to use the pen. The doctor was saying just teach them with the needle. I said no, they are going to get a pen, he has never used a pen, how is he going to do it?  We had to make this patient stay ... I didn’t have this knowledge… (FG3)*  *the pre diabetes stage, I know it’s something new to me, so because I had, you know, I have a family history, my father is diabetic, so I didn’t know, you know, it’s really important to be, you know, to know a little bit more to help, so that I won’t get diabetes for myself. So you know, obviously, if I get this sort of knowledge, I can apply in clinical. Participant Focus Group 2 (FG2)*  *I’ve learnt quite a lot from it, yeah……., I was really surprised on, especially the stats and stuff, …., I’m thinking, wow, I actually do know them quite well, but I learnt heaps from it. (FG1)*  *Difficult. I found it hard. (FG1)*  *I wouldn’t say it was easy because you had to fully focus and read, you had to focus really. (FG3)*  *It was for an endocrinology ward; it wasn’t for a mixed ward. (FG1)*  *You know, diabetic educators, someone who deals with it 24/7. (FG1)*  *I personally found that we probably needed more instruction at the beginning. (FG1)*  *I don’t know enough about this general topic, and that’s when I went and got the other resource.........maybe if we got a bit of information to start with, then you could choose how much you put into it. (FG3)*  *Maybe if you have something to read first like a blurb bit and then you answer questions about it, rather than just answering the questions and hoping you get them right straight up. (FG1)*  *I had a patient who was on insulin previous there, and thank the Lord, I did night shift and I came in the morning, I just saw the patient is, you know, getting the ketones checked and you know, four times, and I knew why it’s being checked and the importance of being checking ketones. I feel like, I feel good because I did this, I did that, I know why they are checking, you know. Participant Focus Group 2 (FG2)*  *I would really do believe in what this is trying to achieve and I would really like to know more, but I did find myself quite frustrated. (FG2)* |
| --- |
| 1. ***Functionality of program/webpage***   *I think, frustrating after a while when you’ve got to do it again and again and it’s still not right, and you go and, you know, whatever the resources are and come back. (FG1)*  *I got frustrated with it, I wanted to throw my computer out of the window at one stage. One particular group of questions…. there were multiple answers and you just get one wrong, and then it takes you back and you have to read the resources again. (FG1)*  *I personally found myself frustrated, repeating questions that I’d already answered correctly Participant Focus Group 2 (FG2)*  *Four options are a little bit hard…. One option would be better. (FG1)*  *I feel disappointed, and so I’m getting everything wrong. (FG2)*  *I found I had to read just to make sure of my answers so that I can proceed to the next one, but then I still fail. (FG2)*  *You’d gone through and you got a few answers wrong, see at the end where it tells your score, what the correct answers are, and you’re like okay, that one’s out, so you go and you read your resources and come back and get the same question and you select what the program has told you the correct answer and it comes back wrong again. (FG1)*  *I understand there is a minimal four answers, did the four answers, so the four answers are really complicated to me, because even these two answer will be better. (FG1)*  *It’s very time consuming, especially like when you’re working and like, our ward is a very busy ward, like doing that, it’s impossible to do that when you are on duty. Plus, when you go home, I don’t know, if you have family life, it’s different. It’s, you have to be focused on what you are doing Participant Focus Group 2 (FG2)*  *We needed more acknowledgment for the time we put in to do the course. (FG1)*  *It takes longer than two hours, that’s what I found…. For me, three hours. Clinicians might appreciate some protected time to do it. (FG2)*  *The two point, whatever, hours it was, was nowhere near, I think it was 2.4 or 2.6 or something. You easily would have done 10 to 12 hours’ worth. (FG1)*  *I personally found the website good. (FG1)*  *I found it easy to understand, it was good to come back into again. (FG1)*  *There was a couple of times when I had clicked submit for like a question, not the whole thing but the question, and then it would have like a loading problem. (FG1)*  *Some of the resources actually didn’t connect through, it was like error codes. And did you have experience of these errors in your home computer?....... No, not mine. I had no problem at all. (FG1)* |
| 1. ***Contextual and Clinical Relevance***   *It’s the timing of the medications, it’s good to know how it works. That to me was, like, how it works, like is it interactive. (FG1)*  *I think it did help you go away and look at stuff and come back, particularly for medications because I’m not giving them all the day. (FG1)*  *The pharmacology was really hard. (FG1)*  *I definitely wouldn’t say quick is definitely the wrong way. I think if you focused for the first module, the first six units, yes, they were reasonably quick, once you got over the trough business, and everything else, that was reasonably clear. When you got into the heavy pharmacy stuff, now way was that. (FG1)*  *Some of it was also decreasing the length of the questions. (FG1)*  *Some of the terminology is different. (FG1)*  *Australian system and UK system is a little bit different…. different insulin (FG3)*  *There were just little tiny threads of it, and I thought this is getting very British again or whatever. (FG1)*  *The person is a hypo, the first intervention was a little bit different compared to the Australian standard… (FG3)*  *And the resources were great to have, and it was great to have the different levels of resources as well. I loved the way they (resources) were graded. (FG1)*  *I just used this resource, which was a good learning experience in itself because now I’ll keep that resource with me when I’m doing my clinical work. (FG2)* |
| 1. ***Perceived Access Barriers and Facilitators***   *The only problem was that, as I said, the contents, it is a lot, you really need all the content but the time should be given… I think it is very important. (FG3)*  *I can tell you as a negative thing, you put so much […family, kids, working full-time, go home, go to sleep. By the time you try to do and you’re counting the hours. (FG3)*  *We have to rush, I have to rush at times, and then I woke up early one day to finish it off before anybody else got up (FG3)*  *I think that making it compulsory … time to do it…… there are so many things to get done. So isn’t there enough time. (FG3)*  *From personal experience with my staff, I dedicate two hours for everything, before they started training or whatever, but it never worked because you always called in, once you start work, you’re called in and kind of stuck, so half a day block off. (FG3)*  *Suppose I did most of mine here, while I was eating lunch, so that constant interruptions, I found that, you know, I was making mistakes because I was being interrupted, that was the only time that I had available. (FG1)*  *It’s not easy because it does take time to sit down and focus. I did try and do some whilst I was on night shift but I ended up becoming so tired, that I was putting in the wrong stuff in. (FG1)*  *It’s very time consuming, especially like when you’re working and like, our ward is a very busy ward, like doing that, it’s impossible to do that when you are on duty. Plus, when you go home, I don’t know, if you have family life, it’s different. It’s, you have to be focused on what you are doing. (FG2)*  *You’ve got to remember there’s only X amount of computers on the floor and on daylight hours, you’ve got all the other staff needing to use it, afternoon and night shift is different, it’s more that you’ve got a chance to use it, but that is the problem and you don’t know what your workload is going to be. I mean, you can be having a wonderful day and it only takes one MET call and that’s it, your day is gone. (FG1)*  *I’m just starting, you know, because you need to concentrate. (FG2)*  *If you make a mistake, obviously you have to go back, and that’s like, you to do the whole thing and it’s a very long process. (FG2)* |
